# 1539. Presence of mental health concerns is associated with poor adherence in people taking pre-exposure prophylaxis in real-world clinical practice in the United States

**DOI:** 10.1093/ofid/ofad500.1374

**Published:** 2023-11-27

**Authors:** Fritha Hennessy, Libby Turner, Victoria Higgins, James Piercy, Tim Holbrook

**Affiliations:** Adelphi Real World, Bollington, United Kingdom, Bollington, England, United Kingdom; Adelphi Real World, Bollington, United Kingdom, Bollington, England, United Kingdom; Adelphi Real World, Bollington, United Kingdom, Bollington, England, United Kingdom; Adelphi Real World, Bollington, United Kingdom, Bollington, England, United Kingdom; Adelphi Real World, Bollington, United Kingdom, Bollington, England, United Kingdom

## Abstract

**Background:**

Pre-exposure prophylaxis (PrEP) is used to prevent HIV acquisition and is up to 99% effective when users are fully adherent. Research has shown an inverse relationship between mental health difficulties and PrEP adherence. This analysis aims to understand the real-world impact of mental health on PrEP adherence in the United States (US).

**Methods:**

Data were drawn from the Adelphi PrEP Disease Specific Programme™, a real-world, study with retrospective clinical data and cross-sectional PROs conducted August 2021-March 2022 in the US. Physicians provided data on patient demographics, satisfaction, components of mental health including anxiety/depression and PrEP adherence. Current PrEP users (PU) provided data on satisfaction and adherence via the Adelphi Adherence Questionnaire (ADAQ), scored from 0 to 4 where lower scores indicate greater adherence. Analyses of variance and descriptive analyses were performed.

**Results:**

PU (n=196) were categorized as having no (nMH, n=108), mild (mMH, n=44), or moderate/severe mental health problems (sMH, n=26). Anxiety (38.6% mMH, 32.3% sMH) and depression (25.0% mMH, 61.5% sMH) were the most common mental health-related comorbidities (physician-stated). PU with sMH had significantly poorer patient-reported adherence than nMH (ADAQ mean [standard deviation] nMH, 0.29 [0.54]; mMH 0.64 [0.59]; sMH 0.78 [0.6]; p< 0.01). The most common reason for poor adherence was forgetting to take PrEP (27.3% nMH; 21.9% mMH; 50.0% sMH; physician stated). A higher percentage of PU with sMH were dissatisfied with the ease of use of their PrEP than PU with nMH (Fig 1A), a lower percentage of PU with sMH felt PrEP was easy to access than PU with nMH (Fig 1B). PU stated drivers of poor adherence included forgetfulness, not being in usual routine, stigma (Fig 2), particularly in PU with sMH.

**Figure:**
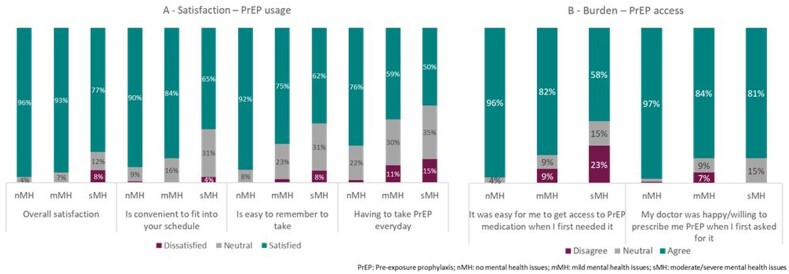

**Figure:**
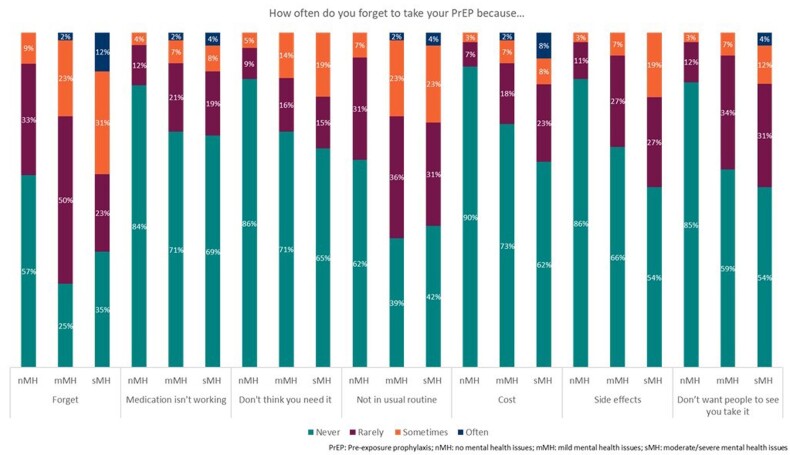

**Conclusion:**

Poorer mental health was associated with lower PrEP adherence, and was a barrier to effective use. Reasons for poor adherence are multi-factorial and not just related to forgetting to take PrEP. Development of PrEP that is easier to use and easier to access will benefit all PrEP users, particularly for people identified as having poorer mental health.

**Disclosures:**

**James Piercy, MSc**, Adelphi: Salaried Employee

